# High-Content Imaging to Phenotype Antimicrobial Effects on Individual Bacteria at Scale

**DOI:** 10.1128/mSystems.00028-21

**Published:** 2021-05-18

**Authors:** Sushmita Sridhar, Sally Forrest, Ben Warne, Mailis Maes, Stephen Baker, Gordon Dougan, Josefin Bartholdson Scott

**Affiliations:** a Cambridge Institute of Therapeutic Immunology & Infectious Disease, University of Cambridge Department of Medicine, Jeffrey Cheah Biomedical Centre, Cambridge, United Kingdom; b Wellcome Sanger Institute, Hinxton, United Kingdom; Teagasc Food Research Centre

**Keywords:** high-content imaging, image analysis, bacteria, antimicrobial resistance, phenotyping

## Abstract

High-content imaging (HCI) is a technique for screening multiple cells in high resolution to detect subtle morphological and phenotypic variation. The method has been commonly deployed on model eukaryotic cellular systems, often for screening new drugs and targets. HCI is not commonly utilized for studying bacterial populations but may be a powerful tool in understanding and combatting antimicrobial resistance. Consequently, we developed a high-throughput method for phenotyping bacteria under antimicrobial exposure at the scale of individual bacterial cells. Imaging conditions were optimized on an Opera Phenix confocal microscope (Perkin Elmer), and novel analysis pipelines were established for both Gram-negative bacilli and Gram-positive cocci. The potential of this approach was illustrated using isolates of Klebsiella pneumoniae, Salmonella enterica serovar Typhimurium, and Staphylococcus aureus. HCI enabled the detection and assessment of subtle morphological characteristics, undetectable through conventional phenotypical methods, that could reproducibly distinguish between bacteria exposed to different classes of antimicrobials with distinct modes of action (MOAs). In addition, distinctive responses were observed between susceptible and resistant isolates. By phenotyping single bacterial cells, we observed intrapopulation differences, which may be critical in identifying persistence or emerging resistance during antimicrobial treatment. The work presented here outlines a comprehensive method for investigating morphological changes at scale in bacterial populations under specific perturbation.

**IMPORTANCE** High-content imaging (HCI) is a microscopy technique that permits the screening of multiple cells simultaneously in high resolution to detect subtle morphological and phenotypic variation. The power of this methodology is that it can generate large data sets comprised of multiple parameters taken from individual cells subjected to a range of different conditions. We aimed to develop novel methods for using HCI to study bacterial cells exposed to a range of different antibiotic classes. Using an Opera Phenix confocal microscope (Perkin Elmer) and novel analysis pipelines, we created a method to study the morphological characteristics of Klebsiella pneumoniae, Salmonella enterica serovar Typhimurium, and Staphylococcus aureus when exposed to antibacterial drugs with differing modes of action. By imaging individual bacterial cells at high resolution and scale, we observed intrapopulation differences associated with different antibiotics. The outlined methods are highly relevant for how we begin to better understand and combat antimicrobial resistance.

## INTRODUCTION

Antimicrobial resistance (AMR) is one of the greatest current challenges in human health, with rising cases of antimicrobial-resistant bacterial infections and a lack of new classes of licensed antimicrobials (1, 2). Advances in bacterial genomics have revolutionized our ability to genotype antimicrobial-resistant bacterial isolates at scale. However, it remains critical to link genotype with phenotype in order to interpret the biological and clinical relevance of AMR. Some phenotyping methods have been adapted to work at scale (e.g., antimicrobial susceptibility testing using semiautomated platforms such as the bioMérieux Vitek system), yet many others either rely on low-throughput methods or aggregate data from mixed populations of bacterial cells. The analysis of bulk bacterial populations rather than individual cells potentially overlooks persister cells or the emergence of resistant or tolerant bacteria within that population. High-throughput imaging of bacterial populations at the scale of individual cells has received limited attention but may be achieved by exploiting high-content microscopy.

High-content imaging (HCI) can be utilized as a powerful phenotypic screening approach that combines automated microscopy with image analysis to quantify multiple morphological features. This approach may capture subtle differences in structure and shape not discernible by the human eye or conventional phenotypic methods. Such image-based profiling has great potential in high-throughput drug screening, which has mainly been applied to eukaryotic cells and tissue (3, 4). In the field of microbiology, HCI has predominantly been used to study intracellular pathogens such as Mycobacterium tuberculosis (5–8) and Salmonella species as they interact with host cells (9) but only recently to screen individual bacteria growing as a population in batch culture (10, 11). Pogliano and colleagues developed a bacterial cytological profiling assay to identify morphological changes in Escherichia coli and other species in response to different classes of antimicrobials using fluorescence microscopy (12–15). Analysis of image data enabled the assignment of distinct morphological profiles correlating with the mechanism of action of the antimicrobial compounds tested (12). This method opened up a novel way of screening new therapeutic compounds simultaneously for efficacy and mode of action (MOA) using bacterial imaging (10–15).

Given the variety of AMR mechanisms harbored by bacterial species and, in many cases, by isolates of the same species, it is important to optimize HCI approaches for a range of bacteria. In this study, we developed and optimized a high-throughput imaging method based on HCI to systematically screen individual bacteria from three different species grown under antimicrobial exposure. We optimized bacterial imaging conditions using an Opera Phenix confocal microscope (Perkin Elmer) and established novel analysis pipelines for image segmentation and bacterial morphological analysis for both Gram-negative bacilli and Gram-positive cocci. The combination of HCI and image analysis enabled the detection of subtle morphological characteristics that differed between different antimicrobial classes. This work contributes to the expansion of microbial phenotyping from population-level to single-cell analysis and provides a comprehensive method of bacterial phenotypic screening at scale.

## RESULTS

### High-content imaging and analysis of individual bacteria.

**FIG 1:**
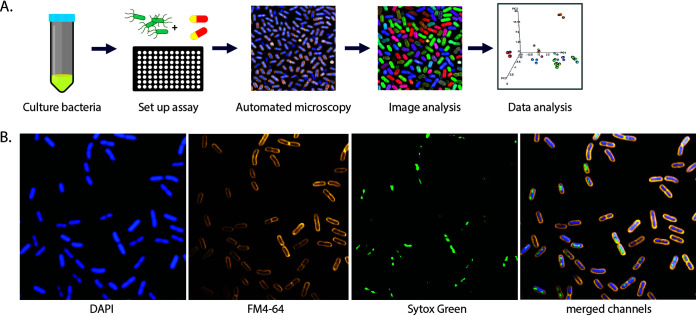
Bacterial high-content imaging. (A) Schematic of the bacterial high-content imaging workflow. Overnight bacterial cultures are added to ultrathin bottom plates and incubated with or without antimicrobial compounds. Adherent bacteria are fixed and stained before being imaged on an Opera Phenix high-content confocal microscope using a 63× water immersion objective. Images were analyzed using Harmony software, and data were exported and plotted in R. (B) Representative image of K. pneumoniae NCTC 43816 stained with FM4-64 (cell membrane), DAPI (nucleic acid, membrane permeative), and SYTOX green (nucleic acid, membrane impermeative).

An HCI workflow was established using two reference isolates from each of the three bacterial pathogens Salmonella enterica serovar Typhimurium, Klebsiella pneumoniae, and Staphylococcus aureus (Fig. 1A; Table 1). Organisms were selected to have contrasting AMR profiles within each species. Each of the isolates was exposed to different antimicrobial agents, and HCI was used to collect phenotypic data for numerous individual bacteria within each assay. To this end, overnight bacterial cultures were grown in 96-well microtiter plates in the presence or absence of each antimicrobial for 2 h to capture multiple early morphological changes. The antimicrobials used are listed in Table 1. To capture images, the bacteria in each well were stained *in situ* with markers for the cell membrane (FM4-64), nucleic acid (4′,6-diamidino-2-phenylindole [DAPI]), and membrane permeability (SYTOX green) (12) (Fig. 1B). Imaging was performed on an Opera Phenix, and image analysis was conducted using Harmony software.

**TABLE 1:** MICs determined by Etest^*a*^

Species	ID	MIC (μg/ml)										
		Ampicillin	Azithromycin	Trimethoprim-sulfamethoxazole	Ciprofloxacin	Gentamicin	Rifampin	Meropenem	Tigecycline	Cefuroxime	Oxacillin	Vancomycin
K. pneumoniae	NCTC 13438	>256*	32	>32*	>32*	6	16	0.125	0.094	>256*	ND	ND
K. pneumoniae	ATCC 43816	64	2	0.5	0.16	1.5	>32*	0.125	0.5	1.5	ND	ND
*S*. Typhimurium	NCTC 13347	0.5	2	0.25	0.012	2	16	0.125	0.19	3	ND	ND
*S*. Typhimurium	NCTC 13348	>256*	3	0.25	0.012	3	24	0.064	0.19	3	ND	ND
S. aureus	NCTC 6571	ND	ND	0.125	0.094	2	ND	ND	ND	ND	0.19	1
S. aureus	ATCC 29213	ND	ND	0.19	0.25	2	ND	ND	ND	ND	0.38	1.5

a * indicates the MIC is above the highest antimicrobial concentration on the Etest; ND indicates that the MIC was not determined.

**FIG 2:**
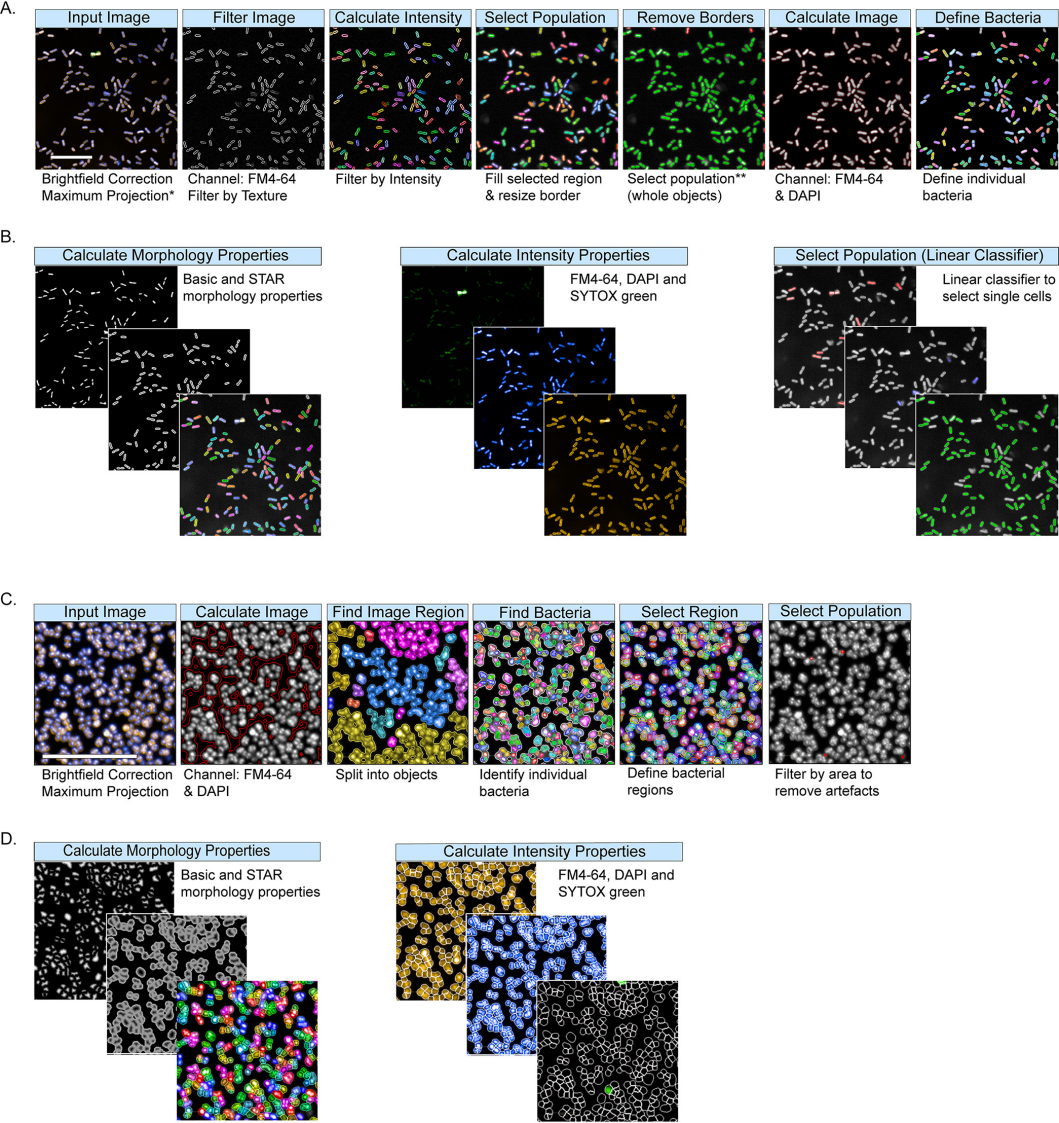
Harmony bacterial image analysis workflow for Gram-negative rods (A and B) and Gram-positive cocci (C and D). (A) Using basic bright-field correction and maximum projection, images were segmented by filtering the images using texture properties based on the FM4-64 channel to remove any background. The image region was filled and resized, and border objects were excluded to include only whole objects. The image region was further calculated using FM4-64 and DAPI fluorescence, and individual bacteria were defined. (*, single planes were analyzed for *S.* Typhimurium; **, left and bottom border object are not displayed as the image is cropped for better visualization). (B) Bacterial morphology and stain intensity properties were calculated using DAPI, SYTOX green, and FM4-64 fluorescence. Finally, a linear classifier was used to train the software to define single bacterial cells and exclude any artifacts. (C) Using basic bright-field correction and maximum projection, the bacterial region was defined using a calculated image based on DAPI and FM4-64 channels. Individual bacteria were identified within the image region, and the bacterial regions were defined and resized into individual bacterial cells. Any artifacts were removed using size filters. (D) Bacterial morphology and stain intensity properties were calculated using DAPI, SYTOX green, and FM4-64 fluorescence. Scale bars shown on input images correspond to 20 μm.

As both rod- and coccus-shaped bacteria were imaged at the single-cell scale, it was necessary to build separate, parallel pipelines for accurate analysis of microorganisms with different morphology. Examples of image segmentation and analysis of Gram-negative rods and Gram-positive cocci are shown in Fig. 2 and detailed in Table S2 at https://doi.org/10.17605/OSF.IO/DC25N, respectively. Images of Gram-negative rods were initially filtered using FM4-64 intensity patterns to enhance stained objects and subtract the background (Fig. 2A). All images were then calculated based on DAPI and FM4-64 intensity and resized to include both cytosolic (DNA) and membrane regions; filtering was performed to remove artifacts such as incomplete bacterial bodies (Fig. 2A and C). Morphological features and stain intensities were calculated for each defined bacterial cell, including area, roundness, width, and length, as well as various measures of the intensity, symmetry, and distribution of each of the FM4-64, DAPI, and SYTOX green channels within each object (Fig. 2B and D). For Gram-negative rods, the segmented objects were further classified as either single bacterial cells, dividing cells, or artifacts (Fig. 2B) with a manually trained linear classifier using Harmony PhenoLOGIC. Subsequent analyses were conducted on the single cells only. The number of individual bacteria captured and analyzed per well was dependent on isolate and treatment but was a minimum of 2,000 bacteria per untreated well for all replicates.

### Optimizing bacterial imaging.

The bacterial isolates displayed different degrees of adhesion to the base of the 96-well plates, which significantly affected the image quality and downstream analysis. For example, the nonmotile K. pneumoniae isolates spontaneously strongly adhered to the bottom of the wells, whereas the motile *S.* Typhimurium isolates displayed relatively poor adhesion, resulting in blurry superimposed images of the flattened z-stack (maximum projection) (see Fig. S1A and B at https://doi.org/10.17605/OSF.IO/DC25N). Consequently, it was necessary to assess multiple plate coating conditions for each *S*. Typhimurium isolate to identify the optimal conditions for binding and image clarity. Ultimately, the image segmentation pipelines were used to quantify individual bacteria and assess staining intensity on 11 commercially available coating matrices (thick and thin rat tail collagen, Matrigel, vitronectin, fibronectin, Cell-Tak, laminin, wheat germ agglutinin [WGA], poly-l-lysine, gelatin, and mouse collagen) in comparison to noncoated wells (see Table S1 at https://doi.org/10.17605/OSF.IO/DC25N).

**FIG 3:**
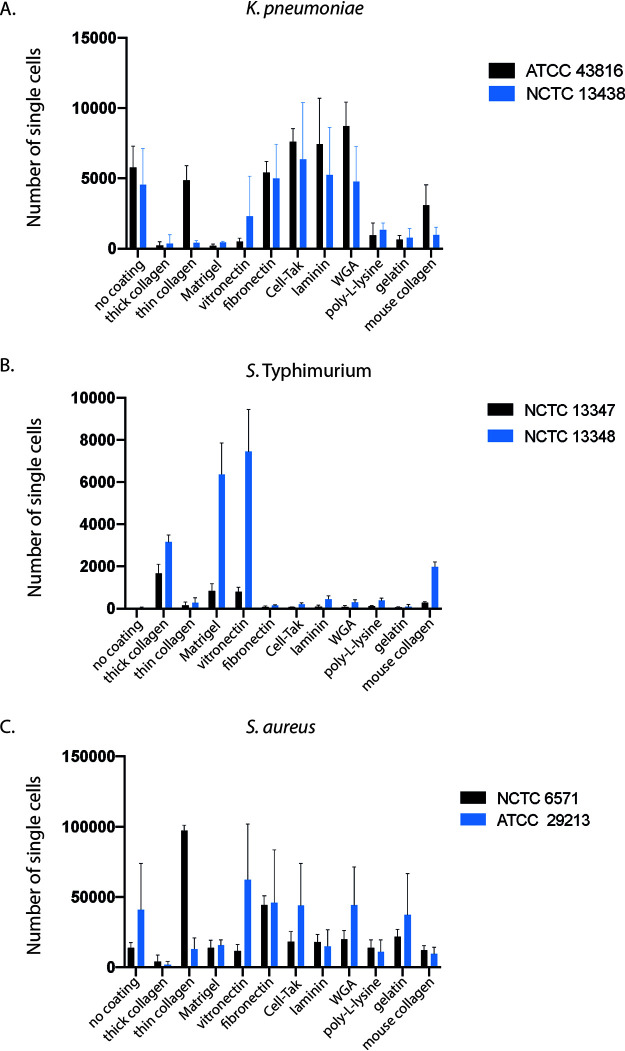
Optimizing plate coating for bacterial adhesion. Isolates were grown in ultrathin 96-well plates on different surface matrices, and the Harmony analysis pipelines were used to count the number of adherent bacteria after fixing, washing, and staining. Graphs are comparing the adhesion of two representative isolates of K. pneumoniae (A), *S*. Typhimurium (B), and S. aureus (C) on each substrate. Error bars represent standard deviations from three biological replicates.

Apart from thick collagen, none of the coating matrices had any noticeable effect on the staining intensities of FM4-64, DAPI, or SYTOX green (see Fig. S2 at https://doi.org/10.17605/OSF.IO/DC25N). However, the optimal coating conditions for adhesion were found to differ between species and, to a lesser extent, for each isolate (Fig. 3). In addition to measuring the number of adhered bacteria, images were assessed for image clarity as well as for any coating-induced morphological differences such as bacterial aggregation (see Fig. S3 at https://doi.org/10.17605/OSF.IO/DC25N). The K. pneumoniae isolates displayed the best adhesion and image quality on noncoated, WGA-coated, and fibronectin-coated plates (Fig. 3A; also see Fig. S3A at https://doi.org/10.17605/OSF.IO/DC25N). Therefore, all subsequent experiments with these isolates were conducted on noncoated wells. In contrast, image quality and the number of adherent *S.* Typhimurium bacteria improved dramatically upon optimization of the plate coating (see Fig. S1C at https://doi.org/10.17605/OSF.IO/DC25N). Both *S*. Typhimurium isolates displayed very poor adhesion to noncoated wells (Fig. 3B; see also Fig. S3B at https://doi.org/10.17605/OSF.IO/DC25N) but adhered sufficiently, although to different extents, to wells coated with thick rat tail collagen, Matrigel, and vitronectin (Fig. 3B; see also Fig. S3B at https://doi.org/10.17605/OSF.IO/DC25N), with collagen and vitronectin being the optimal conditions for NCTC 13347 and NCTC 13348 adhesion, respectively. While different coatings were chosen for the two isolates, it would have been feasible to use the same coating, as the number and image quality of adhered organisms on rat tail collagen, Matrigel, and vitronectin were sufficient for analysis. To overcome any residual lack of adhesion of *S*. Typhimurium, image analysis of these isolates was performed on individual planes rather than a maximum projection of three z-stacks.

The two S. aureus isolates displayed different adhesion properties, with ATCC 29213 showing increased adhesion on vitronectin-, fibronectin-, Cell-Tak-, WGA-, and gelatin-coated wells, whereas NCTC 6571 had sufficient cell counts only on thin collagen- and fibronectin-coated wells (Fig. 3C). Taking adhesion and image clarity (see Fig. S3C at https://doi.org/10.17605/OSF.IO/DC25N) into account, thin collagen and vitronectin were used for optimal adhesion of NCTC 6571 and NCTC 29213, respectively.

### Measuring distinct morphological changes in response to antimicrobial compounds.

**FIG 4:**
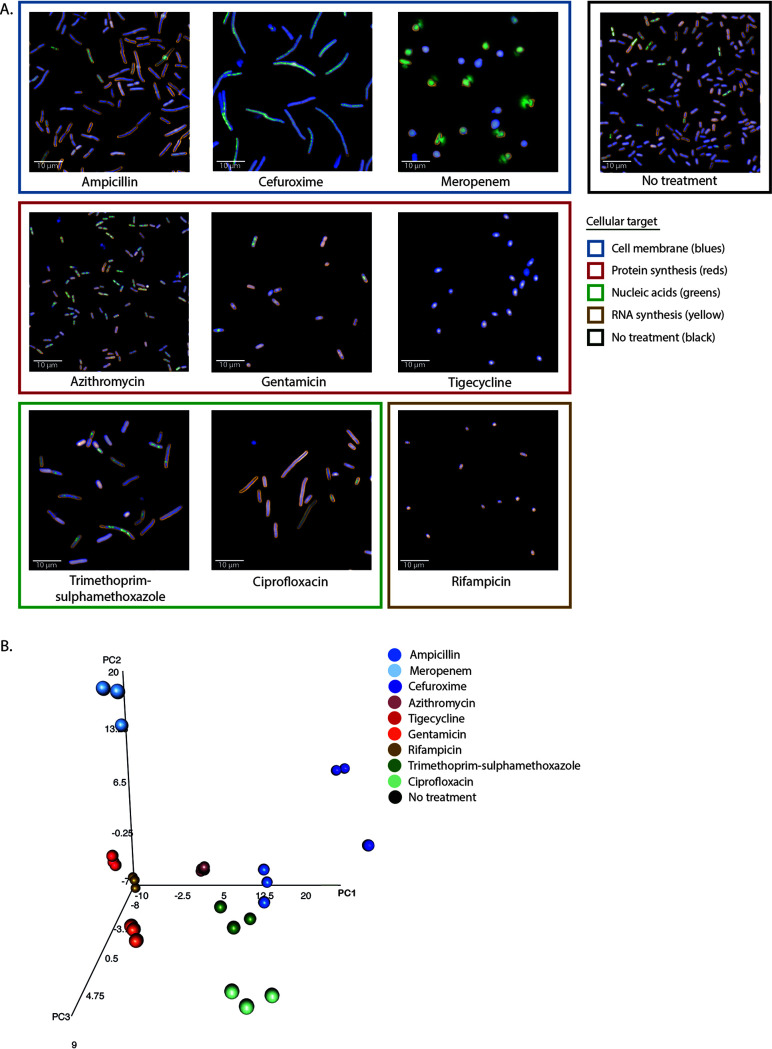
Morphological effects on K. pneumoniae NCTC 43816 under antimicrobial pressure. (A) Representative images of the effect of different antimicrobials on the K. pneumoniae isolate NCTC 43816 in exponential growth phase after 2 h of incubation. Antimicrobials are grouped by similar cellular targets. Bacteria were stained with FM4-64, DAPI, and SYTOX green. Images were acquired on an Opera Phenix using a 63× water immersion lens. (B) Three-dimensional principal-component analysis of the mean and standard deviation values of 95 morphological and intensity properties measured for single bacterial cells in each well. Technical triplicate repeats are shown.

**FIG 5:**
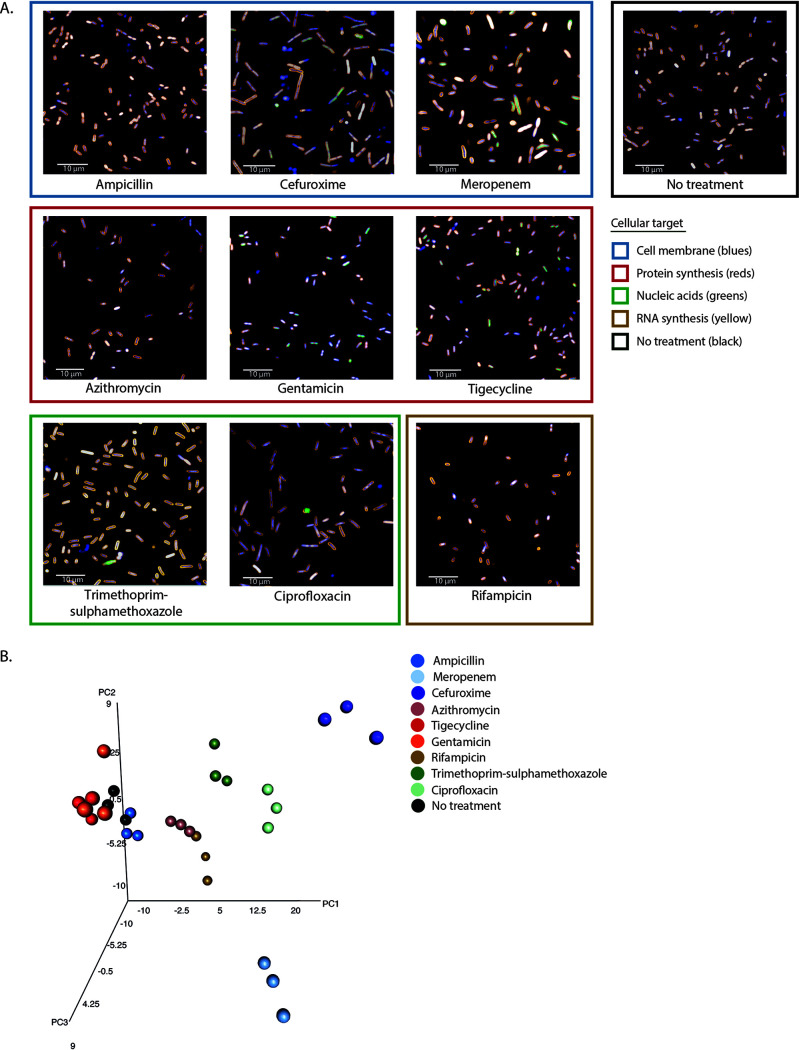
Morphological effects on *S*. Typhimurium NCTC 13348 under antimicrobial pressure. (A) Representative images of the effect of different antimicrobials on the *S*. Typhimurium isolate NCTC 13348 in exponential growth phase after 2 h of incubation. Antimicrobials are grouped by similar cellular targets. Bacteria were stained with FM4-64, DAPI, and SYTOX green. Images were acquired on an Opera Phenix using a 63× water immersion lens. (B) Three-dimensional principal-component analysis of the mean and standard deviation values of 94 morphological and intensity properties measured for single bacterial cells in each well. Technical triplicate repeats are shown.

**FIG 6:**
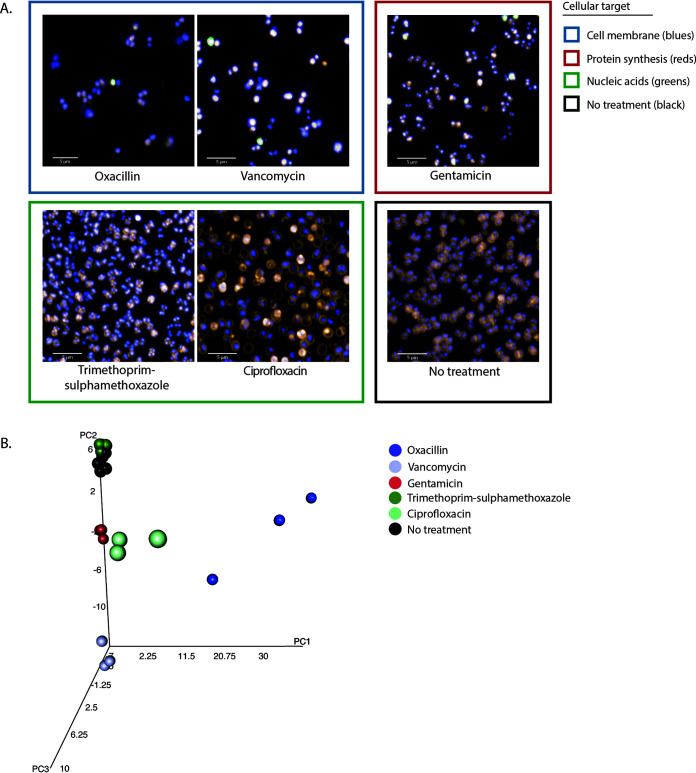
Morphological effects on S. aureus ATCC 29213 under antimicrobial pressure. (A) Representative images of the effect of different antimicrobials on the S. aureus isolate ATCC 29213 in exponential growth phase after 2 h of incubation. Antimicrobials are grouped by similar cellular targets. Bacteria were stained with FM4-64, DAPI, and SYTOX green. Images were acquired on an Opera Phenix using a 63× water immersion lens. (B) Three-dimensional principal-component analysis of the mean and standard deviation values of 92 morphological and intensity properties measured for single bacterial cells in each well. Technical triplicate repeats are shown.

To measure the phenotypic effects of antimicrobials with distinct MOAs, bacteria were incubated with 11 commercially available antimicrobials for 2 h and imaged as described above. Antimicrobials were used at 5× the MIC determined by Etest or 5× the highest concentration tested if an isolate had an MIC higher than the Etest range (Table 1). Figure 4A, Fig. 5A, and Fig. 6A show examples of the observed morphological changes at 2 h posttreatment for a nonmotile (K. pneumoniae NCTC 43816) and a motile (*S*. Typhimurium NCTC 13348) Gram-negative rod and a Gram-positive coccus (S. aureus ATCC 29213), respectively.

There were notable differences that the pipeline was able to capture between the effects of the same antimicrobials on Gram-negative and Gram-positive bacteria, with more visually striking morphological changes observed in the Gram-negative bacteria. The established image analysis pipelines produced mean and standard deviation measurements for >90 morphological features and stain intensities for each bacterium imaged (see Tables S3 to S5 at https://doi.org/10.17605/OSF.IO/DC25N). These measurements were combined for each isolate and analyzed using principal-component analysis (PCA). Technical replicates of each class of antimicrobial separated into distinct clusters based on MOA (Fig. 4B, Fig. 5B, and Fig. 6B; see also Fig. S4 at https://doi.org/10.17605/OSF.IO/DC25N), and biological replicates produced similar distribution by PCA, demonstrating assay reproducibility (see Fig. S5 at https://doi.org/10.17605/OSF.IO/DC25N). Although separate pipelines were required for Gram-positive and Gram-negative organisms, each pipeline was able to distinguish a wide variety of phenotypes generated by antimicrobial treatment, segmenting the images and identifying individual bacteria despite the morphological changes associated with each antimicrobial (see Fig. S6 at https://doi.org/10.17605/OSF.IO/DC25N).

Antimicrobials acting on similar cellular processes generally induced comparable morphological changes in each species, and these were found to cluster in a PCA. Bacteria treated with tigecycline and gentamicin, which block protein synthesis by binding the 30S ribosomal subunit, clustered proximally for all Gram-negative isolates tested (Fig. 4B and Fig. 5B). In addition, these generally also clustered near rifampin- and azithromycin-treated bacteria (Fig. 4B and Fig. 5B); these antimicrobials affect protein synthesis by inhibiting RNA polymerase or translation by binding the 50S ribosomal subunit, respectively. Antimicrobials that inhibit DNA synthesis (trimethoprim-sulfamethoxazole), DNA replication (ciprofloxacin), and cell wall synthesis (ampicillin, cefuroxime, and meropenem) tended to induce an elongated phenotype and again clustered proximally (Fig. 4B and Fig. 5B). Notably, meropenem clustered separately from the other β-lactams for the Klebsiella isolates and appeared to disrupt the bacterial cell wall more potently, causing the bacteria to swell and lyse instead of elongating (Fig. 4; see Fig. S4A at https://doi.org/10.17605/OSF.IO/DC25N). K. pneumoniae NCTC 13438 was resistant to ampicillin, trimethoprim-sulfamethoxazole, cefuroxime, and ciprofloxacin at concentrations higher than the Etest scale, and with the exception of ciprofloxacin, these clustered with the untreated control (see Fig. S4A at https://doi.org/10.17605/OSF.IO/DC25N). A similar phenotype was observed for *S*. Typhimurium NCTC 13348 treated with ampicillin (Fig. 5B). This highlights that HCI screens provide novel data regarding drug susceptibility as well as MOA.

The morphological changes observed for S. aureus were relatively subtle compared to those for the Gram-negative isolates (Fig. 6A). Only ciprofloxacin induced a visually discernible phenotypic change, which was associated with enlarging bacterial area. However, after image analysis, each antimicrobial effectively separated into unique clusters by PCA, except trimethoprim-sulfamethoxazole, which clustered alongside the untreated controls (Fig. 6B; see also Fig. S4C and S5 at https://doi.org/10.17605/OSF.IO/DC25N). This finding suggests that the analysis could discriminate between very subtle cellular variations by capturing and analyzing a large number of phenotypic parameters.

The distribution of variance and the top 50 individual measurements contributing to principal components 1, 2, and 3 are shown in Fig. S7 at https://doi.org/10.17605/OSF.IO/DC25N. These illustrate the relatively small individual contribution of each measured parameter to the principal components, which further highlights the power of analyzing and combining multiple phenotypic measurements.

### Measuring the relative importance of specific morphological and fluorescence intensity parameters.

To assess the quality of the image analysis, we calculated the Z prime (Z′) values using Harmony, comparing treated and untreated bacteria for each species and antimicrobial combination (see Tables S6 to S8 at https://doi.org/10.17605/OSF.IO/DC25N), where an ideal assay should yield values between 0.5 and 1 (16). The Z′ values were higher for antimicrobials that clustered further from the untreated control in the PCA, and the Gram-negative isolates generally had more Z′ values above 0.5 than the Gram-positive isolates. For example, the trimethoprim-sulfamethoxazole-treated S. aureus ATCC 29213 failed to separate from the untreated control by PCA, which correlated with poor Z′ values (see Table S8 at https://doi.org/10.17605/OSF.IO/DC25N). Similarly, poor Z′ values were obtained for azithromycin-treated K. pneumoniae NCTC 43816 (which is intrinsically resistant to macrolides) and gentamicin-treated *S*. Typhimurium NCTC 13348 (see Tables S6 and S7 at https://doi.org/10.17605/OSF.IO/DC25N).

**FIG 7:**
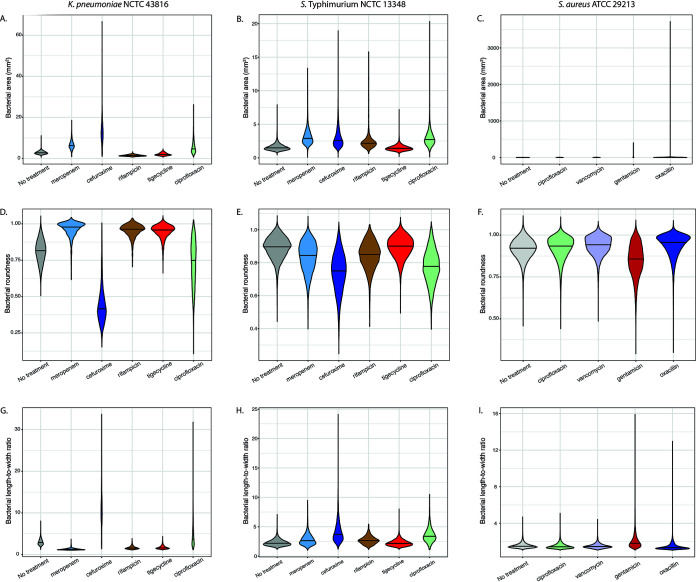
Comparison of individual basic morphological measurements. Violin plots of bacterial area (A to C), bacterial roundness (D to F), and bacterial length-to-width ratio (G to I) comparing K. pneumoniae NCTC 43816 and *S*. Typhimurium NCTC 13348 treated with meropenem, cefuroxime, rifampin, tigecycline, and ciprofloxacin and S. aureus ATCC 29213 treated with ciprofloxacin, vancomycin, gentamicin, and oxacillin, with untreated controls.

To highlight the relative importance of some of the measured parameters with high Z′ values, differences in measurements across representative antimicrobials were compared. Morphological measurements of roundness, area, and length-to-width ratio (Fig. 7), as well as threshold compactness and the radial relative deviation of the DAPI and FM4-64 staining patterns, were plotted for a selection of antimicrobials with different MOAs (see Fig. S8 at https://doi.org/10.17605/OSF.IO/DC25N). In addition, SYTOX green intensity was included as this should stain bacteria only if membrane integrity has been compromised (see Fig. S8M to O at https://doi.org/10.17605/OSF.IO/DC25N).

When plotting these parameters individually, clear differences were observed between the different antimicrobials for the K. pneumoniae isolates and, to a lesser extent, the *S*. Typhimurium isolates. For example, increased area, decreased roundness, increased length-to-width ratio, and FM4-64 and DAPI radial relative deviation correlated with the observed elongation phenotype observed for cefuroxime and ciprofloxacin (Fig. 7A, B, D, E, G, and H; also see Fig. S8A, B, D, and E at https://doi.org/10.17605/OSF.IO/DC25N). In contrast, increased FM4-64 and DAPI threshold compactness as well as bacterial roundness was observed for rifampin and tigecycline (Fig. 7D and E; and see also Fig. S8G, H, J, and K at https://doi.org/10.17605/OSF.IO/DC25N).

Generally, SYTOX green intensity was higher for antimicrobials disrupting the bacterial membrane for both Gram-negative (meropenem) and Gram-positive (oxacillin) isolates. However, the effect of individual parameters on S. aureus was subtler than for the Gram-negative isolates, with only gentamicin treatment showing slightly decreased roundness and increased length-to width ratio (Fig. 7F and I), demonstrating the need to observe multiple combined phenotypic parameters.

### Phenotypes within a bacterial population.

**FIG 8:**
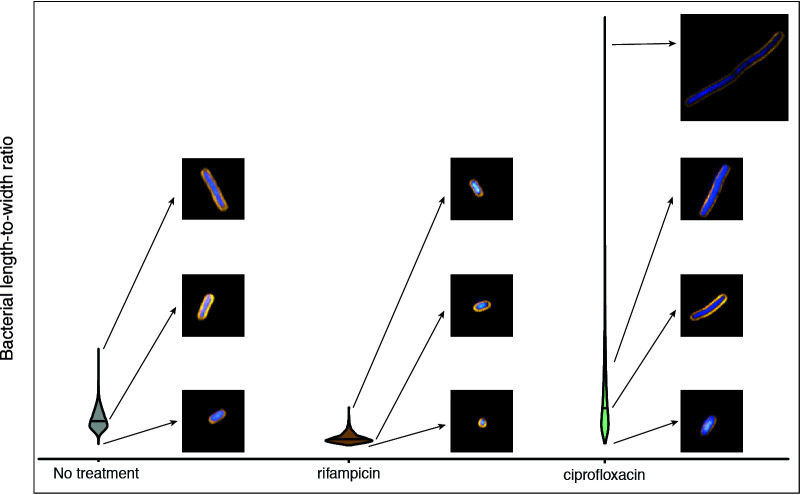
Example of population-level length heterogeneity of K. pneumoniae NCTC 43816. Violin plot of bacterial length-to-width ratio comparing untreated, rifampin-treated, and ciprofloxacin-treated K. pneumoniae NCTC 43816 with inset images demonstrating the different phenotypes observed under the same growth conditions within a single well.

Using violin plots, it was possible to visualize the population density and distribution of bacteria for any given parameter. This analysis demonstrated the inherent morphological heterogeneity in bacterial populations of the same isolate under the same growth conditions (Fig. 7; see also Fig. S8 at https://doi.org/10.17605/OSF.IO/DC25N). For example, certain antimicrobial treatments yielded a high degree of heterogeneity in the length-to-width ratio, notably cefuroxime and ciprofloxacin treatment of K. pneumoniae NCTC 43816 (Fig. 8). In contrast, rifampin treatment appeared to yield decreased variability within a population compared to untreated controls (Fig. 8). By phenotyping single cells, it is possible to observe within-population differences, which is critical for identifying persistence or emerging resistance during antimicrobial treatment.

## DISCUSSION

In this study, we optimized an experimental pipeline for high-throughput confocal imaging of motile and nonmotile bacteria in liquid culture. We used this method for systematic screening of Gram-positive and Gram-negative bacteria under antimicrobial pressure, with robust and standardized image analysis pipelines to efficiently and reproducibly measure distinct morphological changes correlating with antimicrobial MOA. This analysis was built around profiling the subtle morphological phenotypes of individual bacteria in a culture, providing information on the whole population and variation within that population.

There are a number of advantages to using HCI for bacterial research. It provides flexibility in experimental design, with the ability to customize and compare growth conditions and individual isolates from different species in high throughput. Traditional phenotyping methods rely on the collective properties of large numbers of bacteria, but HCI enables measurements at the scale of individual bacterial cells. Advances in image analysis permit reliable segmentation of bacterial images and rapid, detailed profiling of individual bacterial cells with the ability to demonstrate the heterogeneity of bacterial phenotypes in any given environment.

Our work identified some challenges in using HCI for bacterial research, in particular, variation in adhesion to microtiter plates. Poor adhesion influences both image quality and the number of bacteria successfully imaged for downstream analysis. This challenge was overcome by testing a range of coating matrices, which demonstrated substantial inter- and some intraspecies variation in their ability to adhere to each substrate. For example, there were notable differences in adhesion between the nonmotile K. pneumoniae and motile *S*. Typhimurium. K. pneumoniae possesses an array of adhesins that allow it to adhere and persist in different environments, which have contributed to its emergence as an important nosocomial pathogen (17, 18). In contrast, *S.* Typhimurium relies on motility and more specific cellular interactions and invasion for causing infection (9). These factors highlight the need to optimize imaging conditions for each bacterial isolate. It is also important to consider the potential effect different coating matrices may have on bacterial growth, staining patterns, and morphology. In this study, we observed some isolate-dependent bacterial aggregation on different matrices, as well as a slight decrease in staining intensity on thick collagen. However, we were able to optimize adhesion and imaging conditions for all isolates tested, and in most cases, more than one coating condition was sufficient for downstream analysis, making it possible to screen multiple isolates in parallel using the same plate coating for the higher-throughput assays.

One of the most challenging aspects of image analysis was the segmentation and identification of individual bacteria. This is in part because most existing image analysis software is designed primarily to analyze images of eukaryotic cells. However, analysis pipelines to effectively segment both rod- and coccus-shaped bacteria were created using existing image analysis tools in the Harmony software. Though the analysis pipelines in this study were created using Harmony, which is a proprietary software from Perkin Elmer, there are open-access image analysis software options available—for example, CellProfiler (19) and Cellpose (20)—which have similar analysis capabilities.

It was necessary to produce separate pipelines for coccus- and rod-shaped bacteria for the initial segmentation. Other studies have also utilized different pipelines for phenotypically variant species; for example, the analysis used by Zoffmann and colleagues for E. coli was not suitable for Acinetobacter baumannii, as these species differ in size and shape (10). Importantly, the pipelines created in our study could be used to reproducibly segment bacteria under all growth conditions used, even as morphologies changed due to antimicrobial exposure. Distinct morphological changes were observed in response to different classes of antimicrobials, with different effects observed in Gram-negative versus Gram-positive species. However, bacteria from the same species generally displayed similar morphological distributions by principal-component analysis when treated with 5× the MIC, correlating with antimicrobial mechanism. In addition, different clustering was observed between susceptible and resistant isolates, allowing for simultaneous evaluation of potency as well as MOA.

The phenotypic changes identified in this study in the presence of antimicrobials are comparable to previous imaging studies in *Enterobacteriaceae*, including bacterial enlargement with carbapenems and cephalosporins (21, 22), compaction of the nucleoid with antimicrobials targeting the bacterial ribosome (23), and filamentous elongation in the presence of fluoroquinolones (24). In agreement with other studies, we identified similar morphological changes in isolates of K. pneumoniae and *S.* Typhimurium as those previously reported for E. coli in response to a range of antimicrobial classes (12), but here, we employed a simplified method by removing centrifugation steps and by imaging directly in wells rather than on agarose pads. This facilitates higher throughput and scalability.

The fluorescent staining protocol previously optimized by Nonejuie et al. (12) worked well across all the isolates tested in this study. FM4-64 stains the cell membrane, and the staining patterns should relate to membrane integrity. DAPI and SYTOX green both stain nucleic acids, but only DAPI is permeative through an intact cell membrane, making SYTOX green intensity an additional measurement of membrane integrity after antimicrobial exposure (25, 26). In addition, nucleic acid stains can distinguish between subtle alterations in nucleic acid distribution patterns. Plotting individual phenotypic parameters was sufficient when an antimicrobial induced a strong visual phenotypic effect: for example, length-to-width ratio could be used for ciprofloxacin- or cefuroxime-treated K. pneumoniae. However, in most cases, and in particular for the smaller coccus-shaped S. aureus isolates where the phenotypic effects were subtler, a combination of morphological and stain intensities, distribution, and symmetry measurements were required to efficiently evaluate the data. This highlights that the software can detect important variations that are not obvious in conventional phenotypic methods.

Our methods contribute to moving microbial phenotyping from a population-based analysis to the scale of individual bacterial cells and provide a comprehensive method of bacterial phenotypic screening at scale. This approach has a wide range of applications, but the ability to provide analysis of diverse collections of isolates simultaneously under a range of growth conditions gives it important potential in the fight against AMR. In addition to existing roles in compound screening for antimicrobial efficacy and simultaneous MOA prediction (27), the technology could be used for more detailed mechanistic follow-up studies using mutant libraries to assess genes that are protective against individual drugs (28). HCI could also be adapted for high-throughput rapid antimicrobial susceptibility testing of clinical isolates (29). Large numbers of compounds and bacterial isolates, representing species with diverse genetic backgrounds, can be screened at scale. We have previously shown the utility of bacterial HCI for therapeutic antibody screening (11), and there is potential to assess synergy between antimicrobials and monoclonal antibodies against multidrug-resistant bacteria that would be challenging using other platforms. Importantly, by analyzing individual bacteria within a culture, it is possible to detect differential effects and persister cells during drug treatment and be able to truly evaluate the efficacy of a compound.

## MATERIALS AND METHODS

### Bacterial isolates.

A number of reference bacterial isolates, representing clinically important species, were analyzed. This panel included one Gram-positive (Staphylococcus aureus) and two Gram-negative (Salmonella enterica serovar Typhimurium and Klebsiella pneumoniae) species. Two isolates were included per species, each with broadly different antimicrobial susceptibility profiles (Table 1).

### Antimicrobial susceptibility testing.

Antimicrobial susceptibility testing was performed for a range of clinically relevant antimicrobials with different MOAs (Table 1). MICs were determined by Etests (bioMérieux) according to the manufacturer’s instructions. Briefly, pure bacterial cultures were diluted in saline to an 0.5 MacFarland standard, 100 μl of solution was inoculated and spread onto Iso-Sensitest plates (Oxoid; CM0471), and an Etest strip was placed on top. Plates were incubated for 16 to 18 h at 37°C before the result was read.

### Preparation of plate coatings.

Coating matrices were prepared according to manufacturer recommendations under sterile conditions (see Table S1 at https://doi.org/10.17605/OSF.IO/DC25N). All coatings, except poly-l-lysine, were incubated in ultrathin 96-well plates (Perkin Elmer CellCarrier Ultra, 6655308) overnight at 37°C. The following day, wells were rinsed 1 to 3 times with wash buffer (see Table S1 at https://doi.org/10.17605/OSF.IO/DC25N). For poly-l-lysine, wells were coated for 5 min. The solution was aspirated, and wells were left to dry overnight at 37°C.

### Bacterial imaging assay.

Overnight stationary-phase bacterial cultures were diluted in LB broth and mixed with antimicrobials to a final antimicrobial concentration of 5× MIC. Where an MIC could not be measured (i.e., where bacterial growth continued along the whole length of the Etest), the upper limit of the Etest was arbitrarily used in place of the MIC. The bacteria were incubated with and without antimicrobials in static incubators in ultrathin 96-well plates for 2 h at 37°C. The plates were aspirated, and the remaining adherent bacteria were fixed with 4% paraformaldehyde (Alfa Aesar, J61899.AK) for 10 min. The wells were washed once with 50 μl of Dulbecco phosphate-buffered saline (DPBS) (Thermo Fisher, 10010023) before staining. Fixed cells were stained with 50 μl per well FM4-64 (2 μg/ml, Thermo Fisher, T13320), SYTOX green (0.25 μM, Thermo Fisher, S7020), and 4′,6-diamidino-2-phenylindole dihydrochloride (DAPI; 2 μg/ml, Sigma, D9542) diluted in Hanks balanced salt solution (HBSS) buffer (Thermo Fisher, 14175095). Staining was performed at ambient temperature for 20 min in the dark followed by a wash with 50 μl PBS. Finally, 50 μl of PBS was added to wells, and the plates were imaged within 24 h.

### High-content imaging and image analysis.

High-content confocal imaging was performed using an Opera Phenix (Perkin Elmer), using a 63× water immersion lens. Ten fields of view (equating to 0.4 mm^2^) were imaged for each well, with 3 z-stacks per field at 0.5-μm intervals to ensure comprehensive imaging of the bacterial monolayer. Triplicate biological and technical replicates were performed for all experiments. Image analysis was performed using Harmony (v4.9). Optical correction was performed using flat-field and bright-field correction. The detailed full analysis pipelines are shown in Table S2 at https://doi.org/10.17605/OSF.IO/DC25N. Data were exported and plotted in GraphPad Prism and R (30).

### Data availability.

All data underlying the results are available in supplemental Tables S3 to S8 at https://doi.org/10.17605/OSF.IO/DC25N. Associated images can be made available upon request.
